# Corporate Restructuring Laws Under Stress: The Case of Spain

**DOI:** 10.1007/s40804-023-00283-5

**Published:** 2023-05-08

**Authors:** Ignacio Tirado

**Affiliations:** Professor of Commercial Law, Universidad Autónoma de Madrid, Madrid, Spain

**Keywords:** COVID-related legal reform, Institutional reform, Extraordinary economic measures, Private law, Insolvency law, Private over-indebtedness, Public over-indebtedness

## Abstract

The COVID-19 crisis caused an unprecedented global disruption of economic activity, which was especially intense in Spain due to the nature of its economy. Many legal and institutional reforms were adopted, and extraordinary economic measures implemented. As interim reforms are lifted and economic incentives wear off, Spain will need to grapple with the economic damage caused by the pandemic. Arguably, the reform of the insolvency system recently approved, which precedes and is independent of the measures enacted to stave off risks caused by the pandemic, provides an enhanced and improved framework to deal with business insolvency. Spain now counts on a state-of-the-art hybrid restructuring system and a modern regulation to deal with the financial and economic distress of micro enterprises. However, the special legislation approved during COVID, and the side effects of the economic measures came with a cost and are already interfering with the day-to-day application of the new insolvency system, especially concerning public claims and public guarantees. Further, the Spanish legal framework has still some shortcomings which might prove a real hindrance to resuming access to credit at adequate levels.

## Introduction

The COVID-19 health emergency and the Government’s measures adopted to contain it had a very strong impact on the Spanish economy. Due to its high reliance on tourism and its dependence on certain strategic imports, the Spanish economy shrunk by 11.3% in 2020, and recovery, as restrictions were lifted, took longer than in most other countries in the region, with a growth of only 5.5% in 2021 and 2022. Naturally, this negative development has substantially worsened by the increase in the cost of energy and raw materials, as well as by the rapidly spiking inflation caused by the war in Ukraine, and started only when COVID-related hazards were beginning to appease. These two consecutive, exogenous ‘perfect storms’ put Spain’s economy to the hardest, real-life stress test.

Unsurprisingly, Spanish authorities reacted by implementing a comprehensive package of legal reforms and economic measures. Both types of measures must be analyzed together as they have a high degree of joint functionality. The legal measures concerned several areas of the law (e.g., contracts, civil procedure, corporate law, tax law, labor law, and, obviously, restructuring and insolvency law); economic measures included financing facilities, subsidies and investment projects. This paper seeks to offer a comprehensive—and critical—overview of the main measures included in the package, as well as an identification and consideration of possible lessons that can be extracted from—arguably—one of the most widespread, simultaneous economic disruption the world, in its global dimension, has ever lived through.

## Legal Measures

The pandemic forced the Spanish Government to adopt strict measures limiting the mobility of persons, severely hampering the development of many economic sectors. As a direct or indirect result, contracts were interfered with, exports and imports were reduced, services remained unperformed, and, generally, businesses saw their activity drastically reduced or even completely halted. In light of this unprecedented, almost dystopic reality, *exceptional* legislation was passed. The measures implemented through the COVID-19 special legislation were limited in time and scope. They sought to preserve the going-concern value of the economy, freezing ordinary remedies for non-compliance, avoiding effects envisaged for financial distress, and facilitating financing to businesses. Once the emergency situation passed, exceptional rules would be—and mostly have been—removed with it.

### Exceptional Rules Affecting General Areas of the Law

Labor law was one of the first areas of reform. The reduction of business activity brought about a parallel decline in the need for labor alongside a decrease in business revenues. In order to protect employment, special legislation was passed.^1^ The most relevant reform consisted in temporarily deactivating the legal system that regulated the termination of employment by considering dismissals based on pandemic-related reasons as non-justified. Instead, a new system of temporary suspension of employment contracts was introduced,^2^ with partial relief for social security duties for the employer and direct subsidies to employees. This mechanism allowed job preservation, limiting costs for businesses and curbing social damage.

Another area where taking urgent legislative action was considered necessary concerned procedural law. Direct or indirect interference with contractual performance was one of the immediate consequences of the COVID measures, and the contract law institution of *force majeure* and the court-defined hardship rule (implicit clause *rebus sic stantibus*) would not suffice to avoid wholesale litigation and the clogging of the already overburdened Spanish courts. Naturally, the rules were again limited in time and scope. Generally, enforcements and executions were suspended for part of the time of the duration of the state of emergency, with the period of suspension of foreclosure on mortgage loans lasting longer. The duty for the vulnerable to pay rent was also temporarily suspended, as were evictions. But the more important reform concerned the use of technology. Remote procedural acts by the parties were allowed for many types of procedures, and the technological capacity of the court system was substantially enhanced. Digitization, modernization and an enduring institutional upgrade of the Spanish court system have been among the few positive externalities generated by the emergency.

Emergency legislation was also enacted in the remit of company law.^3^ In this area, a substantial enhancement of the use of technology was already underway when COVID arrived, and additional measures to facilitate distant functioning of companies and their related governance were approved, some of which were envisaged to last beyond the emergency context.

But companies as debtors were the main focus of the pandemic-related reform. According to ordinary Spanish company law, when the company’s capital has fallen below 50%, shareholders must either remedy the situation or dissolve the company. The rule, aimed at avoiding insolvency—and externalizing excessive risk on the market – by requiring mandatory action at a time when the company is still balance-sheet solvent, was suspended for debtors falling under the 50% threshold following losses incurred in 2020 and 2021.^4^ Obviously, the measure sought to prevent the closure of viable, solvent but distressed companies. In line with this rule, and in a more controversial fashion, the reform also allowed for the possibility to update the value of assets in the books, otherwise a limited possibility based on ordinary financial accounting principles (i.e., prudence); and, also with a view to preserving the continuation of businesses, the reform included the temporary suspension of the possibility to terminate subordinated shareholder loans linked to benefits (*préstamos participativos*).^5^

### Exceptional Rules Affecting Insolvency Law

The core of the reform—and the part most relevant to this paper—concerns emergency rules included in the insolvency legislation.^6^ The rules can be clustered into three different types, depending on their finality: (i) those aimed at avoiding the mandatory or forced opening of insolvency proceedings to ensure both that viable but COVID-stressed businesses did not undergo unnecessary—value-destructive— proceedings and that the court system did not suffer a sudden increase in cases; (ii) those aimed at facilitating access to finance for a debtor, at a time when credit was expected to be scarce; and (iii) rules seeking to protect ongoing proceedings or bolster general rules of the system in light of the challenges posed by the emergency.

(i) An ‘insolvency moratorium’ was introduced with the first emergency legislation and was later extended. The moratorium entailed, as of the declaration of the state of emergency (14 March 2020), the inability for creditors to successfully attain the declaration of formal insolvency proceedings (*concurso de acreedores*) for a period of time whose final date was 31 December 2021. Technically speaking, creditors could file for the insolvency of their debtors, but their petition would not be given procedural course. Conversely, debtors’ ability to file for the opening of insolvency remained untouched; moreover, if a creditor had filed for insolvency earlier, a petition by the debtor would take priority and insolvency would be guided by the rules of voluntary filing (e.g., the debtor would, in principle, remain in possession, as opposed to its substitution by an insolvency representative when insolvency is declared following a creditor’s filing). Perhaps more importantly, the debtor’s duty to file for insolvency within a period of 2 months from the moment it—or its directors— knew or should have known of the situation of insolvency was suspended.^7^ The suspension of this duty, breach of which could entail civil liability and civil sanctions, was extended first until 31 December 2021, and later until 30 June 2022. The practical relevance of this suspension is apparent since the duty to file is linked to the situation of ‘insolvency’, which, under Spanish law, includes both balance-sheet and, especially relevant in this case, cash-flow insolvency (i.e., the debtor’s inability to pay its debts as they fall due).^8^
(ii) The need to facilitate access to credit by financially troubled debtors was addressed by the suspension of the rule that provided for the automatic subordination of financing granted by ‘insiders’ (shareholders/group companies, directors, family members). This exception would affect financing given to the debtor in a period of 2 years counting from the moment of the declaration of the state of emergency. Unfortunately, the rule expressly states that those claims are to be deemed ‘ordinary’ claims, which is a junior class just above subordinated creditors, and therefore, on the face of it, not enough to make financing sufficiently attractive, even by insiders, who, by definition, already have a stake in the debtor`s viability. The literality of the text would not seem to allow for an interpretation which treats secured claims provided by insiders as privileged for the value of the collateral.
  It is noteworthy that Spain, unlike other countries (e.g., Germany), did not need to introduce a special rule to protect financing from *ex post* avoidance actions, since it was considered that protection is sufficiently granted through the application of its general insolvency framework.^9^
(iii) A number of rules were implemented to protect ongoing insolvency plans and out-of-court refinancing agreements, so as to prevent defaults caused by the exceptional situation from interfering with the conclusion and implementation of procedures that were underway at the time of entry into force of the legislation. Paradigmatically, debtors implementing an insolvency plan or an out-of-court refinancing agreement were given the possibility to present amendments to the plan, and the debtor’s duty to request liquidation or petitions by creditors based on the default of the plan’s implementation was temporarily suspended, provided that certain requirements were met. Further, several measures were included to facilitate liquidations and make on-line sales more efficient and streamlined.

## Economic Measures

Alongside legal reform, Spain introduced, sequentially as the situation made it necessary and European funds were secured, a comprehensive package of economic measures, which included both direct subsidies and investments, as well as indirect wholesale financing programs. This paper will only address the indirect measures because these—apart from being much larger in value—have a clearer connection with legal reform and with the economic situation ensuing after the pandemic.^10^

### Indirect Economic Measures Concerning COVID-19

As part of an EU-wide recovery plan,^11^ Spain designed and implemented an unprecedented public financing scheme to ensure enough liquidity was pumped into the market. The scheme consisted of a line of public guarantees that would secure loans granted through the banking sector, so that the system could avail itself of the expertise and lending network of private banks. The risk was partially shared between the public guarantor and private financial creditors, so that the interests of banks and that of the public ‘lender’ (*Instituto de Crédito Oficial*–ICO) would be aligned. The risk distribution of loans granted was 80% for ICO and 20% for the lending bank in the case of SMEs, and 70% for ICO and 30% for the financial entity, when the debtor was a larger business. The scheme was fully executed, with a total of over 100 billion euros in liquidity loans and over 40 billion euros in investment loans.^12^ The vast majority of borrowers, of both credit lines, were sole entrepreneurs (*empresarios autónomos*) and micro, small and medium enterprises (MSMEs) (90% of total funds, in 74% of the loans) pertaining to all sectors, albeit with a clear predominance of those areas of the market more directly affected by COVID measures, i.e., tourism, entertainment and culture.^13^

The financing scheme featured *ex ante* coordination between the Spanish Government and the banking regulatory institutions, reflected in banks being forced to provision a relatively high percentage of their exposure from the onset. Further, banking exposure as a consequence of the scheme is limited, given the envisaged risk allocation. Of the 140 billion euros, 107 billion constitutes exposure of the public sector, and only 33 billion exposure of the private banks.

Loans were granted with payment exemption periods. Further, as time passed and the crisis loomed, the Government approved additional measures to soften the situation of debtors, allowing them to either: (i) request an extension of the maturity of the loan, with a maximum of 10 years;^14^ (ii) convert the loan into *préstamos participativos*, a type of subordinated loan, with repayment linked to the debtor’s positive financial result in the yearly financial accounts, and not counting as debt for the purposes of capital requirements; or (iii) propose a partial write-down of the loan. Acceptance of the request to adhere to options (i) or (ii) was left to the banks, which had little discretion to reject them if the relatively loose requirements were met;^15^ option (iii), however, requires that stricter conditions are met, and the amount of funds allocated by the Government to write down debt is limited (3 billion euros).^16^ In order to write down the debt, the debtor must renegotiate with the bank, and, among other requirements, prove that revenues during 2020 fell at least by 30% as compared to 2019 if the write-down is to be of an amount up to 50%; and 70%, if the haircut is to reach 75%. There is already data on the use of these options: the option to obtain a rescheduling of payments was requested, successfully, for debts accounting for around 50% of the total money guaranteed; and the option to convert loans into subordinated, benefits-linked loans was hardly used.^17^ The write-down option is available until June 2023, so any data would be anecdotal.

### Indirect Economic Measures Relating to the War in Ukraine

When the pandemic-related measures were lifted and economic recovery was, slowly, starting to kick in, international turmoil generated, directly or indirectly, by the war in Ukraine caused a sudden increase in the cost of raw materials and energy, with inflation spiking in a manner that had been unprecedented for decades. This situation pushed the Spanish Government to implement an additional financing scheme, in many ways similar to the one previously implemented for the COVID-induced crisis.

The program is to be channeled through the private banking network, and will consist, again, of a line of public guarantees (by ICO) for up to 10 billion euros. It is aimed mostly at sole entrepreneurs and SMEs, and its concession is linked to, amongst other requisites, proof of a sharp increase in costs of energy and raw materials.^18^

## The Situation After Legal Reform and Economic Measures

As of December 2022, the situation in Spain would, on the face of it, seem to remain under control. Although numbers concerning insolvency cases have started to increase, they do so slowly.^19^ Social unrest seems limited, and economic indexes are within normality. Exceptional legal and economic measures would seem to have staved off, in the short term, the risk of an immediate socio-economic crisis, notwithstanding the enormous challenge posed by a crisis of unprecedented contours. Furthermore, some of the legislation enacted, especially reforms concerning the institutional framework, has the potential to generate positive effects well beyond the current crisis. There is—or should be—no going back in terms of enhanced use of IT systems, for both companies and courts, and the changes adopted to streamline procedural law ought to remain in place. The use of crises to bolster efficiency and enhance institutional capacity are, indeed, positive lessons learned.

There are, however, reasons for concern. The relatively contained number of insolvency cases and limited increase in litigation can be explained as a direct consequence of the legal and economic measures set out above, the majority of which, in the case of the former, have been lifted, and which, in the case of the latter, can be regarded as temporary solutions. The number of insolvencies is likely to increase considerably as availability of financing is reduced and existing loans become due. The 2 years of non-payment exemption granted to many MSMEs, which are now coming to an end, the temporary suspension of employment contracts (ERTEs), and the secrecy of out-of-court restructurings have the potential to be concealing the actual lack of viability of many enterprises as well as higher figures for unemployment and soon-to-be formal insolvencies. Moreover, the suspension of the duty to file for insolvency or of creditors’ ability to trigger insolvency cases is no longer in place, and, as economic ‘steroids’ are disappearing and the activation of ordinary insolvency rules resumes, the real economic situation will inevitably emerge.

The Spanish experience also shows that there are negative consequences to introducing widespread exceptional legal measures. Exceptional legislation does not come without costs. One type of cost is direct damage to legal certainty. This hazard, of very negative consequences for investment and growth, happens due to hyperinflation of legal changes: the new rules, which constitute exceptions to the ordinary legal framework, were introduced in a spree of legal reforms in a brief period of time, further eroding the understanding of the system. Amendment after amendment was approved as the socio-economic situation evolved, affecting almost all legal sectors. And emergency drafting is ‒ as the Spanish experience shows—a direct enemy of good technical drafting, and hence of legal certainty. Further, institutions and relevant stakeholders must incur considerable transaction costs to both understand and adjust to the new legal framework.

Spain also presents a good example of how exceptional legislation can interfere, *ex post*, with ordinary legislation. This is the case with the legal treatment of credits guaranteed by the public lender (ICO) in an *ex post* out-of-court restructuring or formal insolvency procedure. The regulation is fragmentary and unclear in several aspects. Apart from that, two rules stand out. On the one hand, ICO’s claims against the debtor are to rank *pari passu* with any new financing provided to the debtor, and the public claim cannot be crammed down by creditors in a debt restructuring agreement (in or out of court). The regulation also envisages that any collateral granted to secure future financing should also benefit any previous public ICO claims. This is already proving a serious hindrance to restructuring the debt of viable but financially troubled debtors, makes new financing difficult, and hence jeopardizes the viability of businesses whose chances of survival depend on additional credit being provided. Further, there are limitations to the transfer/assignment of credits which had a public guarantee, since it requires consent from the public creditor and potential purchasers must meet certain requirements that would normally exclude distressed assets funds. This limitation makes credits much less liquid and poses additional problems for banks and, ultimately, debtors.

## The Framework for Dealing with a Post-Crisis Scenario in Spain

As can be inferred from the previous sections, Spain’s approach to dealing with the sudden, exogenous crises suffered in the past 2 years has consisted in providing liquidity and facilitating access to credit for businesses, underpinned by the enactment of substantive exceptional legislation. Perhaps the main direct consequence of this approach has been a dramatic increase in private indebtedness as well as a potential surge in public debt. In this section, I will briefly describe the legal tools available to deal with the situation.

### Dealing with Private Debt Post-COVID

#### The ‘New’ Insolvency Framework

The surge in private indebtedness is happening precisely when the country counts on a renovated insolvency framework. After more than 2 years of work, the Law Commission (*Comisión General de Codificación*) convened to transpose the Restructuring Directive^20^ approved a draft insolvency amendment bill which went much further, and proposed an overhaul of almost the entire existing insolvency system. After a public discussion and several amendments during parliamentary review (which substantially worsened the original text), a new Law was passed on 5 September 2022 (Law 16/2022).^21^ Notwithstanding some evident flaws, the final text constitutes an important effort at the modernization of the system, and, if adequately implemented, it has the potential to become an important instrument for dealing with the ensuing over-indebtedness problem.

The whole Spanish system is regulated in one very lengthy legal text^22^ which is structured in four separate sections (called ‘Books’), one for each type of procedure: section 1 regulates a classic in-court, formal insolvency procedure (*concurso de acreedores*), is the most detailed part of the law, and, with almost 600 articles, includes rules which are applicable generally to all procedures; section 2 regulates restructuring agreements (*acuerdos de restructuración*), hybrid procedures with very limited court intervention; section 3 introduces a new procedure for micro businesses (*procedimiento especial para microempresas*); and section 4 regulates the private international law rules applicable to proceedings with an international element.^23^

Concerning possible exits, the Spanish insolvency system allows for all possible solutions: piecemeal or going-concern liquidation, out-of-court restructuring agreement, in-court restructuring agreement, and a simplified procedure for the discharge of individual debtors. When the debtor seeks to reach an agreement with its creditors (of any kind), the system envisages a ‘negotiation period’ of 3 months, extendable for three additional months (except in the case of micro businesses) where a general moratorium applies, both to enforcement of security rights (provided certain requirements are met, and subject to exceptions) and to early termination clauses (*ipso facto* and related covenants). The use of this ‘negotiation period’ is obviously not mandatory, and debtors are free to negotiate an agreement without statutory ‘shields’.

##### In-Court, Formal Insolvency Proceeding

The classic formal insolvency procedure (*concurso*) has, to some extent, been modernized. An attempt has been made to shorten the lengthy procedure, the system’s most important flaw, and improved rules on the use of insolvency professionals, the approval of the plan (e.g., the introduction of a best-interest-of-creditors test), and the streamlining of liquidation proceedings have been envisaged. This formal procedure allows for either an insolvency plan between the debtor and creditors, or a liquidation, although both exits are not internally consistent (the treatment of some creditors is not the same in the plan voting system and in a waterfall liquidation), nor is the voting system in the plan equal to the system envisaged for hybrid restructuring agreements, the procedure’s natural alternative. These differences create value-destructive incentives for strategic selection of the exit and procedure.

Perhaps the most important feature of the reform lies in the introduction of a pre-pack liquidation, following the models existing in the UK or The Netherlands. In essence, an insolvency practitioner is appointed to look for potential buyers of the business as a going concern; once a buyer is found, insolvency proceedings are commenced and 15 days are provided to receive additional bids. The sale will be concluded inside formal insolvency proceedings, but with a much quicker procedural track. Although this design of the pre-pack substantially improves the previous regulation, there are dubious policy choices which can endanger its use in practice, such as, for example, the buyer’s obligation to continue with the business activity for 2 or 3 years, depending on whether the preferred bid to sell the business is presented at the time of the filing for insolvency or at a later moment. Another positive element of the reform, which substantially improves the previous framework, is the introduction of an express procedure in case of debtors with no or very few assets. Unlike in previous practice (i.e., simultaneous opening and closure of the procedure) or in other countries (e.g., Germany, where a high percentage of insolvency proceedings are never started due to the insufficiency of the insolvency estate to cover the costs), a case apparently without assets will undergo a quick procedure: publicity will be given to the opening of insolvency proceedings, including only reference to the outstanding debt, and creditors amounting to a minimum of 5% can request the appointment of an insolvency practitioner that will look into the affairs of the debtor with a view to discovering potential avoidance of transactions or preferences or possible liability of directors. If nothing is found, the procedure will end. These rules, aimed at allowing a quick, orderly end of economically distressed businesses, are most welcome and can help alleviate the existing backlog of cases, an endemic problem of Spain’s court system.

All in all, and although progress has been made, Spain’s *concurso de acreedores* remains its old self, a classic formal insolvency procedure of a past generation. It is still overregulated and excessively procedural, and its design continues to follow too closely the rigid model of other countries, e.g., Germany (in part, the Spanish legislator’s inspiration in 2003), which, due to their superior institutional framework, are better equipped to deal with rigid procedures. To some of the old flaws, new errors have been added. Perhaps the most evident one is the upgrading of public creditors in the system, not just in terms of hierarchy of claims, but, especially, through the creation of procedural privileges which are certain to cause substantial damage to the system.

##### Hybrid Restructuring Agreements

The *acuerdos de reestructuración* regulated in section 2 of Spain’s insolvency law is meant as the ‘star’ solution to deal with viable but financially distressed businesses. The previous system included several hybrid procedures, which have now been merged and rationalized into just one. The most direct antecedent of the new hybrid procedure, called *acuerdos de refinanciación*, proved a relatively successful instrument in the aftermath of Spain’s severe economic and banking crisis, and, since it was already almost fully compliant with the Restructuring Directive, changes have been limited. The legislator has sought to preserve the best of the previous system, introducing a few rules that international, comparative experience has showcased as best practices, and extending the procedure potentially to all creditors (the previous version only concerned financial creditors).

It is a flexible, efficient hybrid procedure where most of the agreement is negotiated and channeled out of court. Following the example of the UK Schemes of Arrangement, court intervention is, in principle, limited to *ex ante* approval of the formation of classes, when requested (it is voluntary), and to ratification (*homologación*) of the plan, following creditor approval. Conceived as a solution to deal with pre-insolvency situations, it can also accommodate deeply financially distressed debtors as long as the going-concern business is viable in the mid or long term. As with the previous system, non-insolvent debtors may want to resort to this procedure—instead of a purely contractual out-of-court agreement—in order to benefit from protection against *ex post* avoidance, reduce risks of *ex post* liability, provide strengthened protection to creditors ready to finance the implementation of the agreement, or, most importantly, bind dissenting creditors. The procedure can also effectively work as a de facto liquidation of the debtor—not of the business—by including the sale of the business or of business units.

The content of the agreement concerning the restructuring of the business has no limits beyond those set by the general legal framework. Moreover, exceptional rules are envisaged concerning corporate law (e.g., facilitating the approval of structural modifications of the corporation—mergers, splits, etc.—or removing creditors’ right to oppose these measures), contract law (e.g., regarding assignment of contracts), or labor law (e.g., binding decisions adopted outside labor law courts).

The content of the agreement concerning the restructuring of debts is also flexible and mostly in accordance with international best practice. Following the Directive, all impaired creditors must be given a vote, and only a few types of creditors must be left unaffected by the plan (due to social reasons, e.g., certain family law-related claims, tort claims, and, most importantly, labor claims), or only be subject to very limited effects (e.g., public claims). Beyond those exceptions, all creditors—including secured ones—may be affected by the plan, in accordance with a class voting system. There is flexibility as to the formation of classes, as long as creditors of the same class have an objective ‘commonality of interest’, an open concept which has as a requirement that creditors in the same class have equal economic value, as determined by their ranking in liquidation.^24^

The approval of a restructuring agreement requires certain—usual—majorities within each class (2/3 for unsecured classes, 3/4 for secured creditors). Further, as a general rule, all classes must approve the plan. As an exception to this general rule (which will likely be the most frequent case), an agreement can be deemed approved by a simple majority of classes, so long as at least one of these classes is integrated by creditors more senior than ordinary unsecured creditors in liquidation; or by at least one class of creditors that is ‘in the money’, namely, that would presumably receive at least partial payment considering the—expert—valuation of the business as a going concern. In this regard, Spain’s system thus seems to follow the somewhat contradictory rule included in the Directive, mingling two alternatives with little in common.

The new law also includes an inter-class cramdown mechanism with an absolute priority rule (hence following the Directive’s ‘exception’, not the rule), softened, as is the case for many other European countries (e.g., The Netherlands, France, Germany), with a deviation from absolute priority where certain conditions are met, in this case when ‘it is indispensable to ensure the viability of the business, and the claims of affected creditors are not damaged in an unjustified manner’ [Art. 655.3 Insolvency Act (IA)]. Shareholders are treated as a class, and hence may also be crammed down. It must also be noted that secured creditors that have voted against a plan when its class has voted against the restructuring cannot be subject to cramdown in the secured part of their debt. In Spain, protection of secured creditors is thus very high under the new system. Individual creditors, of any type, enjoy basic safeguards, such as a best-interest-of-creditors test, which is construed as what the creditor would have received in a liquidation, piecemeal or as a going concern. Evidently, under Spain’s new system, valuations will become essential. To be sure, if the market is able to adequately cater for the likely demand of such a professional service at a reasonable cost, and if it proves not to be a cause for widespread litigation, the system may work; otherwise, it will not.

The new regulation includes relatively shy measures to incentivize the provision of credit in the period leading to the agreement and later, during its implementation. On the one hand, interim financing and new financing, provided certain requirements are met, will be deemed post-commencement claims for 50% and privileged claims for the rest, in case the debtor ends up in formal insolvency proceedings as a consequence of the failure of the restructuring plan. Furthermore, interim and new financing will be protected from *ex post* avoidance actions, provided there is no proof of fraud, only when the plan was ultimately approved by creditors and ratified by the court, and the financing represents more than 50% of the total outstanding debt in insolvency (more than 60% if the creditor is an especially related person, i.e., an ‘insider’).^25^

In short, it can be stated that Spain’s hybrid restructuring agreements system constitutes a state-of-the-art procedure, a positive development which has the potential to improve the already acceptable results of its preceding scheme. The success of this framework, especially to the extent it is capable of attracting market participants to using it instead of formal insolvency proceedings, can prove instrumental in liberating resources for the courts and increasing legal certainty in a time when credit and investment are likely to require an accommodating legal framework.

However, there remain some uncertainties. The very limited ability to subject public claims to restructuring, especially in a time when they are bound to increase due to the use of public credit to alleviate the crisis, casts a shadow over the efficacy of the reform; and this is bound to be a bigger problem the smaller the business, since public claims tend to be a lesser concern for larger debtors and enterprise groups. Another cause for concern is the rather conservative approach applied to interim and new financing, with limited *ex post* protection and priority in liquidation. At a time when credit is likely going to be scarce, the current regulation might prove insufficient to attract financing for distressed but viable businesses. One additional problem might derive from the moot institutional capacity to deal with the likely increase of business valuations, something exacerbated by the insufficient development of a secondary market for distressed businesses, a question of the essence not only for going-concern sales, but also, obviously, for valuation in the context of restructuring agreements.

##### The Special Procedure for Micro Businesses

Perhaps the most relevant innovation of the recent Spanish reform is to be found in the introduction of a specific, stand-alone, exclusive procedure for micro enterprises. The legislator went beyond what the implementation of the Restructuring Directive required, and decided to tackle what constitutes, arguably, the country’s most pressing regulatory issue: how to deal with the financial and economic distress of the vast majority of businesses, especially in light of the extremely negative results presented by the existing legislative framework.^26^

The main problem of the previous framework was one of disproportionate relative cost. In order to deal with this, the new system has implemented (i) a procedural flexibilization, and (ii) a procedural and functional simplification.

One of the most original elements of the special procedure is its flexible approach to the effects of the opening of proceedings. Unlike in traditional systems, where certain effects, on debtors, creditors, contracts, etc., happen automatically *ex lege* with the opening of the procedure, the Spanish system, conscious of the costs that many of these effects bear for the relevant parties, makes them optional. Labelled the ‘modular approach’,^27^ the system creates two basic, fast-track procedural schemes for both a plan and a liquidation, leaving it to the parties to add ‘modules’ (i.e., procedural effects) as they see necessary, based on a cost-benefit analysis. Hence, for example, an insolvency practitioner will only be appointed at the request of the debtor or—more likely—creditors, and as long as there are funds—from the estate or from the petitioner, depending on the situation—to pay the professional’s fee; or a stay on enforcement of security rights will only be applied if certain circumstances accrue, if and when it is expressly requested. The modular system as designed by the Spanish legislator furnishes parties with enormous flexibility, since they can have a fully-fledged, classic insolvency procedure (almost identical to a *concurso de acreedores*), with professionals, moratorium, debtor removed from management, etc., or, rather, a basic scheme where a plan is discussed or a liquidation implemented with hardly any procedural activity. It will be up to the parties to define the procedure they need on a case-by-case basis. The system is thus based on the parties’ self-responsibility and proactivity.

The new special procedure for micro businesses also features a strong procedural simplification. Unlike the general in-court insolvency procedure, which, following the German model, includes a rigid sequence of procedural stages (common stage followed by a plan or liquidation stage, or a combination of the last two), the general determination of rights and liabilities, the negotiation and vote of a plan, or the liquidation, all take place in parallel, where possible. Inefficient and costly organs like the assembly of creditors have disappeared as such, and most procedural acts are performed by written submissions. Orality is an exception, and it is allowed only when a certain procedural act (i.e., proof) can only take place through a hearing. When hearings are deemed necessary, they will be conducted through electronic means. In fact, technology plays a vital role in the design of the special procedure for micro enterprises.^28^ All written submissions are made through pre-determined, online free templates, and channeled through a protected platform which ensures immediate reception and allows for the tracing, in real time, of procedural acts. Case management is greatly simplified, and the potential for errors, court passivity, or other types of malfunction ‒ relatively common until now—is substantially reduced.

The special procedure for micro businesses seeks to achieve a functional simplification as well. Perhaps the most salient example is the default rule which allows the debtor in possession to liquidate its assets, so long as no creditor requests the appointment of an insolvency professional to do so in its stead. This is paired with the creation of an online liquidation platform, with rather original characteristics. The liquidation platform not only is an online auction website, but also works as a permanent online catalogue, where anyone (not just those that wish to participate in an auction) can purchase assets which have been included by debtors and insolvency practitioners from the different insolvency estates. Moreover, this platform is also tasked with performing liquidation functions even after the procedure has been closed.^29^ Another highly innovative rule is the possibility to have a tacit judicial confirmation of the plan: once creditors and the debtor have agreed, intervention by a judge who validates the plan based on an analysis on the merits will only take place if it is expressly requested by the parties that have not voted in favor. Otherwise, the passing of time will convalidate the plan.

This special procedure foresees a number of rules aimed at tackling issues specific of the insolvency of the smallest debtors. By way of example, section 3 of the Insolvency Law provides a direct link—following a failed plan or a liquidation—to the system of debt discharge for individual entrepreneurs. The law also seeks to address one of the most frequent problems of these cases: creditor passivity. The system is designed to sanction inaction and passivity through a number of rules. For example, many of the notifications must be made directly by the parties through the IT program, and failure to comply penalizes the defaulting party. Further, a ‘scream or live with it’ rule is envisaged, according to which passivity before the issuance of certain documents or adoption of certain decisions waives the interested party’s right to challenge them at a later moment. In line with this, the Spanish system also includes a ‘deemed consent’ rule, which counts a creditor’s lack of vote as a positive vote in case of a plan to continue with the business activity.^30^ Another rule that can prove highly relevant in the insolvency of micro businesses is the adoption of a modified relative priority rule. Unlike in section 2’s restructuring agreements, a plan in the context of the special procedure for micro businesses follows the Restructuring Directive’s default relative priority solution, with a possible equity control by the judge in case a dissenting—allegedly prejudiced—class challenges the plan. Many MSMEs will be family businesses, and often the personality of the debtor and related parties will be key to the survival of the business. In this context, the enhanced flexibility provided by the relative priority rule could prove helpful.^31^

Notwithstanding all the promising features of the new procedure, again some doubts still remain on the horizon. First, the timing of the different procedural stages and actions is too optimistic. This may result in widespread non-compliance by the courts and/or participating insolvency practitioners, which would defeat the entire purpose of the reform. These limited times for liquidation may push the debtor or the insolvency practitioner to rushed, value-destructive fire sales of the assets, although this risk is substantially taken care of through the use of the liquidation platform. Another element of doubt is the adequate functioning of the technology implemented to handle the entire system. The new software, designed in a short period of time, might soon be put to a hard test if insolvencies of MSMEs start to increase, as seems likely. It remains moot how well and fast the court system and the relevant stakeholders will adapt to an overhauled infrastructure.

Again, as in sections. 1 and 2 of the Law, arguably the main problem of the system envisaged in section 3 is the erroneous treatment of public claims. This mistake is especially worrying in the case of MSMEs because they tend to have the—proportionally—largest amount of public claims in their balance sheets. Moreover, it is MSMEs that have most often resorted to the public financing scheme post-COVID, and even in a higher percentage (98%) it is the smallest debtors that have requested a rescheduling of their repayment dates, so ICO claims are likely to be their largest indebtedness going forward. The special procedure for micro businesses has introduced several procedural privileges in favor of public creditors, with the potential to destroy the going-concern value of these businesses in most proceedings. It seems clear that, should a crisis arise, the law will need to be amended, or the private sector will suffer a strong crowding-out effect in the market.

#### Other Non-Insolvency Rules

In the previous section the argument was made that the post-COVID scenario finds Spain with a new, improved insolvency framework, but also, in part, with some doubts as to its implementation and efficacy. However, in order to properly deal with a difficult economic situation in the aftermath of the two consecutive crises (COVID and war-related high inflation, especially of relating to the prices of raw material and energy), more legislative action might seem appropriate especially if a reduction of private indebtedness is to be achieved.

So far, non-performing loans (NPLs) of the banking sector have remained stable.^32^ However, as measures are lifted, they are likely to increase. As explained earlier, the exposure directly assumed by the banking system would seem manageable, and much has already been provisioned. However, given the enormous amount of financing pumped into the system (140 billion euros in little more than 1 year), a growth even in publicly guaranteed debt may end up causing a knock-on effect in other entrepreneurs and ultimately in the banking sector. It is unclear whether Spain’s banking system counts on the necessary tools to deal with a sudden surge in NPLs. Since the real estate crisis, Spain has a ‘bad bank’ in place (SAREB), which may be used for this type of crises, although the sectors involved—and their lack, in many instances, of collateral—in this potential new crisis may make the current design and accumulated experience of little use. If the worst scenario becomes reality, Spain may want to remember the good results achieved with its regulatory treatment of NPLs in the late stages of the recent banking crisis. Until the rule was removed as a result of the request of the European Banking Authority, reaching a refinancing agreement with a debtor was enough to reclassify—upwards—a loan. It was not necessary for the plan to be implemented for a period of time to test its validity. This type of regulatory forbearance, on the face of it risky for banks as debtors, proved an enormous incentive for banks to enter into restructuring agreements. Perhaps if a new state of emergency ensues, consideration ought to be given to temporarily reintroducing this rule.

In a more general fashion, access to credit must be reinforced in Spain, especially for MSMEs. A reduction in the availability of credit, especially in an environment of high interest rates (as is the current situation), might require an improvement in the existing framework to reduce risk for lenders and hence facilitate access to credit. There is plenty of room for improvement in the framework of asset-based financing, especially with regard to the system of secured transactions over movable assets, with special focus on receivables financing, ultimately the only source of collateral for the smaller debtors.

In any case, Spain’s ability to navigate out of murky waters will depend on the level of maximization of the equation sovereign solvency-banking debt-private sector debt.

### The Public Debt Problem

Spanish public debt has been constantly growing since 2010, with a steep increase in 2012 caused by Spain’s financial sector crisis, but with steady yearly increments in the past few years. Numbers now show that Spain is one of the most indebted European countries, with a debt to GDP ratio bordering on 120%.^33^ With such level of indebtedness, and unlike in 2010, when its public debt was hardly 60% to GDP, the country has little buffer to manage an additional increase.

Seen in detail, COVID-related measures imply a total financing scheme of 140 billion euros, with a public Spanish agency assuming more than 75% of that amount. Although a large portion of those funds comes from long-term budget programs of the European Union, they do not come, in any case, without a cost, since the EU has borrowed those funds from the market and they must be repaid. European funds are to be spent on productive uses, aimed at increasing competitiveness and ensuring sustainability in economic growth; they cannot be directly used to cancel NPLs of MSMEs. Thus, in the short term, a sudden increase in NPLs is bound to cause damage to Spain’s capacity to finance itself.^34^ Moreover, a general crisis of the non-financial economy might eventually hit the banking sector too. This would increase financing problems for Spain’s Government, since the contagion channels between banking and sovereign over-indebtedness spread quick and are difficult to contain.

In 2012, Spain and the EU managed to contain the eurozone sovereign debt crisis. But the problem then was different. While in the euro crisis a handful of countries had either excessive public indebtedness (e.g., Greece, Portugal) or excessive financial sector indebtedness (e.g., Ireland, Spain), in 2023 many eurozone countries will have excessive public indebtedness alongside non-financial private sector over-indebtedness, which—as stated—is likely to spread fast to the financial sector. The European Stability Mechanism has—arguably—tools to deal with public sector and financial sector indebtedness, but perhaps not for widespread private sector distress. Further, the current firepower of the ESM may be insufficient, especially considering that this crisis is affecting not just a few countries, but, in different degrees, all of them. The current high amount of liquidity combined with the high level of inflation considerably reduces the ESM’s and ECB’s general ability to react effectively. This dimension of the crisis must be given careful consideration. And the outlook is rather bleak.

## Footnotes

1 The aim of the reform was to avoid the destruction of jobs in viable businesses whose need to reduce labor costs was merely due to the effects of the pandemic. However, separating these businesses from those whose financial distress was caused by other reasons is an extremely complicated matter, difficult to regulate and more difficult to implement. Moreover, markets are interrelated and intertwined, and measures dictated to stave off contagion risks had widespread knock-on effects in almost all sectors of the economy. Vetting of businesses trying to identify those worthy of protection proved impossible in reality, and exceptional legislation was applied across the board.

2 The measure was achieved through the creation of the so-called ERTE (*expediente de regulación temporal de empleo*), which allowed employees to receive 70% of their salary for 6 months, and 50% thereafter. The program, as initially devised for COVID cases, ended in April 2022. The measure seems to have rendered positive results. See, for example, Laborda et al. (2021); Martín et al. (2022).

3 See Royal Decree-law 8/2020, of 17 March, available at: https://www.boe.es/diario_boe/txt.php?id=BOE-A-2020-3824.

4 According to Spanish Company Law, directors who do not take the required action in time can be made jointly liable with the company for debts incurred after the breach of the duty to act (Art. 367 *Ley de Sociedades de Capital*).

5 Consistent with some of the economic measures included in the emergency package, the Spanish reformer sought to protect liquidity of business by providing a certain degree of tax relief. These measures were very limited in number and content, comprising temporary, early measures to postpone and fraction some payments, as well as more direct relief in some economic sectors.

6 Emergency legislation has been fragmentary, and changing over time, with several amendments to implemented measures, and the extension of certain effects. With direct effect on the insolvency legislation, see Royal Decree-law 8/2020, of 17 March, available at: https://www.boe.es/diario_boe/txt.php?id=BOE-A-2020-3824, later amended by Royal-Decree-law 16/2020, of 28 April (https://www.boe.es/buscar/act.php?id=BOE-A-2020-4705 confirmed by Law 3/2020, of 18 September; Royal Decree-law 34/2020, of 17 November, available at: https://www.boe.es/buscar/act.php?id=BOE-A-2020-14368 and Royal Decree-law 5/2021, of 12 March, available at: https://www.boe.es/boe/dias/2021/03/13/pdfs/BOE-A-2021-3946.pdf.

7 Spanish insolvency law foresaw a general moratorium period to negotiate an out-of-court workout aimed at avoiding insolvency, and which, as a general rule, would last 3 months. When that time had lapsed without an agreement, the debtor that was then – still or already – insolvent would need to file for insolvency within 2 months. This duty was also suspended by the COVID legislation.

8 For the contours of this rule under Spanish law, see Tirado (2012). It is noteworthy that, unlike in Germany, the suspension of the duty to file is not expressly limited to situations of insolvency deriving from the COVID crisis. For a comparative overview of the German and Spanish COVID-related reforms, with critical considerations, see Madaus and Arias (2020).

9 See Arias and Madaus (2020), p 339.

10 In March 2021, the Government approved Royal Decree-law 5/2021, which included a line of direct subsidies to sole entrepreneurs and micro enterprises for a total amount of 7 billion euros. These direct subsidies were subject to strict—though reasonable—requirements, and recipient businesses were given plenty of flexibility in using the funds. However, its channeling through the regional institutions, and slow and cumbersome red tape created difficulties in the program’s implementation, which, in the end, was not fully executed. Other programs, better executed, made available for SMEs, were implemented this time through a public financial institution. Other facilities, halfway between direct and indirect measures, were made available to businesses: loans with repayment linked to borrowers’ positive financial performance in the fiscal year, with indebtedness not counting as debt for insolvency purposes; a subsidized, no-interest loan program (6 plus 4 years) for tenants to pay the rent; and other, socially or export-oriented, direct economic financial assistance.

11 See https://commission.europa.eu/strategy-and-policy/recovery-plan-europe_en (accessed 26 February 2023).

12 Liquidity loans were aimed at financing short-term liabilities and the working capital, although plenty of flexibility was envisaged; investment loans were granted to finance expenditure in adaptation, for new equipment, for resuming activities, etc. For detailed regulation, see Royal Decree-law 5/2021, of 12 March, and three regulatory orders (*Acuerdos del Consejo de Ministros*): i.e., of 12 May 2021, https://www.boe.es/boe/dias/2021/05/13/pdfs/BOE-A-2021-7908.pdf, of 30 November 2021, https://www.boe.es/boe/dias/2021/12/01/pdfs/BOE-A-2021-19802.pdf, and of 29 March 2022, https://www.boe.es/boe/dias/2022/03/30/pdfs/BOE-A-2022-4977.pdf.

13 According to the official data published by the Spanish ICO, 1,020,575 liquidity loans were granted to 619,206 borrowers, for a total of 92,880 million euros, which, considering that guarantees only covered 70 or 80% of the loans, means that businesses had access to over 122,377 million euros for liquidity financing; as to investment loans, official figures show 171,909 loans to 130,897 borrowers, 81% of which were sole entrepreneurs or MSMEs; the total amount guaranteed for the investment line was over 14.3 billion euros, for a total financing received of over 18.3 billion euros. Source: Instituto de Crédito Oficial (ICO) (2022).

14 The Decree of March 2020 established a maximum maturity of 5 years and a non-payment period of 12 months; in the Decree of November 2020, debtors were allowed to request an extension of 3 additional years, and one more year of non-payment. Finally, in March 2021, the maximum period was extended to 10 years.

15 In order to convert a loan into a subordinated, benefits-linked loan, the following conditions must be met: the bank agrees based on the adequacy of the business case (‘soft’ gatekeeping: debtor may ‘appeal’); there can have been no default as of the date of application for the loan; revenues must have decreased more than 30% in 2020 with respect to those of 2019; and there must have been a negative financial result in 2020.

16 The write-down will be shared with the bank. In order to be granted a write-down, the requirements set by the Code of Good Practice (*Código de Buenas Prácticas*) must be met.

17 See Instituto de Crédito Oficial (ICO) (2022), p 16 et seq. 98% of debtors that requested the extension were sole entrepreneurs or SMEs.

18 For more detail, see ICO’s official website: https://www.ico.es/en/l%C3%ADnea-de-avales-real-decreto-ley-6-2022-de-29-de-marzo (accessed 26 February 2023).

19 According to the professional association of economists of Spain (*Consejo General de Economistas*), the number of insolvency cases in Spain will increase by 20% compared with 2021, a year when the number of cases had grown by around 50% compared with the previous 5 years. Numbers, however, have remained relatively low compared with the peak reached in 2013, in the aftermath of the financial crisis in Spain, with over 9000 cases. See Atlas (2022).

20 Directive (EU) 2019/1023 of the European Parliament and of the Council of 20 June 2019, https://eur-lex.europa.eu/legal-content/en/TXT/?uri=CELEX:32019L1023.

21 Available at: https://www.boe.es/buscar/doc.php?id=BOE-A-2022-14580.

22 The formal name is Integrated Text of the Insolvency Law (*Texto Refundido de la Ley Concursal*), but is referred to as Insolvency Law in this article.

23 For an analysis of the system before the reform, see Tirado (2012).

24 There are, however, exceptions to flexibility (Arts. 623 and 624 IA): public creditors must be grouped in the same class, as must secured creditors, insofar as the different nature of their rights and value of the collateral justify the formation of separate classes. A separate class must be formed for creditors that are SMEs when the plan requires a write-down of more than 50% of the value of the claim.

25 See Art. 665 et seq. IA.

26 Under the system prior to the reform, fixed and overhead costs of a procedure were similar independently of the size of the debtor. The amount and type of work expected from the machinery of the court system in the formal insolvency procedure of a debtor with 5 employees and 500,000 euros in debt did not differ greatly from that of a medium-sized business. Similarly, the complexity of a small case could, at times, be higher for smaller debtors, with less and worse information available, than for more sophisticated insolvent entities.

27 For a description of this approach, see Davies et al. (2018). This approach has also been considered in the work of UNCITRAL: https://uncitral.un.org/sites/uncitral.un.org/files/media-documents/uncitral/en/msms_insolvency_ebook.pdf (accessed 26 February 2023).

28 The majority of countries with well-functioning insolvency systems have incorporated technology to streamline and facilitate the procedure. By way of example, the use of open-access templates is available in the USA, https://www.uscourts.gov/forms/bankruptcy-forms (accessed 26 February 2023), and in the UK, https://www.gov.uk/government/collections/bankruptcy-and-insolvency-forms (accessed 26 February 2023), amongst many others. It is also in line with the EU approach, see https://ec.europa.eu/commission/presscorner/detail/en/ip_20_2246 and https://ec.europa.eu/info/policies/justice-and-fundamental-rights/digitalisation-justice_es (accessed 26 February 2023).

29 One of the biggest problems of small insolvency cases in Spain was the lengthy—and costly—liquidations, which would go on for years. The new system – perhaps too optimistically—includes a rule that mandates closure of the procedure in maximum 4 months. When the time has passed, the debtor, or the insolvency professional, as the case may be, will send a list of remaining (unliquidated) assets and a list of creditors with outstanding debts to the liquidation platform. For a period still to be determined, the liquidation platform will effectuate periodical payments to the listed creditors, in the order determined, as assets of the catalogue are being sold.

30 The rules of ‘scream or live with it’ (also called ‘scream or die’) and ‘deemed consent’ have been proposed in several instances: e.g., in academia, see Davies et al. (2018), p 63 et seq., or standard-setting bodies, see UNCITRAL, Draft text on a simplified insolvency regime, Deemed approval, para. 18, https://documents-dds-ny.un.org/doc/UNDOC/LTD/V21/009/05/PDF/V2100905.pdf?OpenElement.

31 Unfair or economically unsound solutions can be avoided by the judge’s ultimate control, as well as by the design of the best-interest-of-creditors test, which compares the treatment of each creditor with what they would have received in a going-concern liquidation. On this rule, see Stanghellini et al. (2018), ch. II.

32 Bank of Spain, https://www.bde.es/webbde/es/estadis/infoest/a0403.pdf (accessed 26 February 2023).

33 A comparison can be found at https://datosmacro.expansion.com/deuda (accessed 27 February 2023). Although the debt level is relatively low compared to Japan, USA or Italy, it is undoubtedly a heavy burden for a country with fewer resources than the former.

34 Admittedly, this seems far from an immediate risk, as current numbers show a sharp increase in the demand of Government bonds, even at the retail level. This is caused by the very low remuneration of banking deposits in comparison with the interest rate offered by Government paper. This might, however, be a temporary mirage, and the growing number of retail creditors of sovereign debt could make the potential crisis worse.
